# Inhibition of microbial production of the malodorous substance isovaleric acid by 4,4ʹ dichloro 2‐hydroxydiphenyl ether (DCPP)

**DOI:** 10.1002/mbo3.1174

**Published:** 2021-04-09

**Authors:** Sonja Mayer, Menno Hazenkamp, Martin Kluttig, Dietmar Ochs

**Affiliations:** ^1^ BASF Grenzach GmbH Grenzach‐Wyhlen Germany; ^2^ BASF SE Ludwigshafen Germany

**Keywords:** body malodour, isovaleric acid, laundry care, L‐leucine, *Staphylococcus*

## Abstract

Human body malodour is a complex phenomenon. Several types of sweat glands produce odorless secretions that are metabolized by a consortium of skin‐resident microorganisms to a diverse set of malodorous substances. Isovaleric acid, a sweaty‐smelling compound, is one major malodorous component produced by staphylococci with the skin‐derived amino acid L‐leucine as a substrate. During wearing, fabrics are contaminated with sweat and microorganisms and high humidity propagates growth and microbial malodour production. Incomplete removal of sweat residues and microorganisms from fabrics during laundry with bleach‐free detergents and at low temperatures elevate the problem of textile malodour. This study aimed to analyze the inhibitory effect of the antimicrobial 4,4ʹ dichloro 2‐hydroxydiphenyl ether (DCPP) on the formation of isovaleric acid on fabrics. Therefore, GC‐FID‐ and GC–MS‐based methods for the analysis of isovaleric acid in an artificial human sweat‐mimicking medium and in textile extracts were established. Here, we show that antimicrobials capable to deposit on fabrics during laundry, such as DCPP, are effective in growth inhibition of typical malodour‐generating bacteria and prevent the staphylococcal formation of isovaleric acid on fabrics in a simple experimental setup. This can contribute to increased hygiene for mild laundry care approaches, where bacterial contamination and malodour production represent a considerable consumer problem.

## Graphical abstract

4,4ʹ dichloro 2‐hydroxydiphenyl ether (DCPP) is an antibacterial compound that, when used during laundry, prevents microbial growth on fabrics. This study investigated the potential inhibitory effect of DCPP on the staphylococcal formation of the malodorous substance isovaleric acid in a simple experimental set‐up.

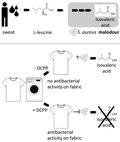

## 1 INTRODUCTION

In humans, the generation of body malodour is attributed to microbial activity on the skin. Various sites of the body harbor diverse glands secreting a wide variety of odorless compounds. This setting represents an attractive environment for a unique composition of microorganisms that transform odorless secretions into malodorous substances (Shelley et al., 1953).

The human body bears three types of sweat glands: The eccrine sweat glands are responsible for thermoregulation and mainly secrete a diluted salt solution and organic compounds such as lactic acid, vitamins, glucose, urea, and amino acids (Huang et al., 2002). The sebaceous glands release lipids and esterified fatty acids, lubricating and waterproofing skin and hair. Finally, apocrine glands found in the human axillae, areolae, genitalia, and ear canal, but not on feet, produce lipid‐ and protein‐rich secretions and odorless steroids (Leyden et al., 1981). These secretions, as well as degradation products of skin‐derived keratin (e.g. from callus; Holland et al., 1990), represent a source of nutrients and water for microbial propagation and serve as a substrate for the production of malodorous compounds. Key species in body malodour are represented by the genera *Staphylococcus, Corynebacterium, Propionibacterium, Micrococcus*, and *Brevibacterium* (Costello et al., 2009; Leyden et al., 1981; Marshall et al., 1988; Minhas et al., 2018; Shehadeh & Kligman, 1963; Taylor et al., 2003).

The odorous substances comprise thioalcohols (sulfanylalkanols), mainly produced by corynebacteria and to a lesser extent staphylococci (Bawdon et al., 2015; James, Austin et al., 2013; Natsch et al., 2004), with 3‐methyl‐2‐sulfanylhexan‐1‐ol (3M3SH) as the most prominent substance (Hasegawa et al., 2004; Troccaz et al., 2004). Furthermore, 16‐androstene steroids, such as 5α‐androst‐16‐en‐3‐on and 5α‐androst‐16‐en‐3α‐ol, mainly biotransformed by corynebacteria, contribute to malodour, however to a low extent (Austin & Ellis, 2003; Gower et al., 1994).

Finally, carboxylic acids were identified as important substances in body malodour. Medium‐chain (C6–C10) carboxylic acids comprise, for example 3‐methyl‐2‐hexenoic acid (3M2H) (Natsch et al., 2006; Zeng et al., 1991) or 3‐hydroxy‐3‐methyl‐hexanoic acid (HMHA) (Natsch et al., 2003) and are mainly attributed to *Corynebacterium* species (James, Austin et al., 2013; Leyden et al., 1981). Short‐chain (C2–C5) carboxylic acids, also termed volatile fatty acids (VFA), such as isovaleric acid, butyric acid, isobutyric acid, indole, 3‐methylindole are predominantly produced by staphylococci (James, Austin, et al., 2013; James, Casey et al., 2004; James, Hyliands et al., 2004; Leyden et al., 1981). The VFA isovaleric acid (IVA) was identified as one of the key components in body malodour exhibiting a distinct cheesy, acidic smell (Caroprese et al., 2009; James, Casey, et al., 2004; James, Hyliands, et al., 2004; Kanda et al., 1990; Leyden et al., 1981). In several comprehensive studies, James et al. have demonstrated how IVA is microbially produced on human skin: Amino acids originating either from sweat secretions or from microbial keratin degradation are used as a substrate for IVA formation. Keratin degradation is accomplished by microorganisms, including *Staphylococcus epidermidis*, propionibacteria (Holland, 1993), or *Kytococcus sedentarius* (Holland et al., 1990, 1992; Longshaw et al., 2002; Nordstrom et al., 1987). The latter was shown to degrade keratin‐containing callus by several keratinases into peptides and free amino acids, for example, L‐leucine. Numerous staphylococci isolated from human axillae and feet metabolized L‐leucine in a semisynthetic medium that mimics human sweat, to 2‐oxoisocaprioic acid, isovaleryl‐CoA and eventually IVA. Besides L‐leucine, also L‐isoleucine and L‐valine are microbially degraded to malodorous VFA, namely, 2‐methylbutyric acid and isobutyric acid, respectively, but to a much lesser extent. (James, Cox et al., 2013; James, Hyliands, et al., 2004).

**FIGURE 1:**
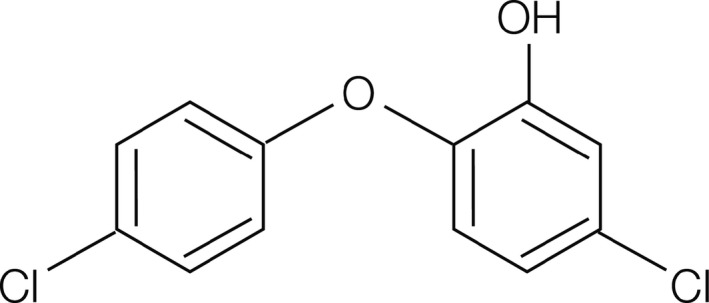
Structure of 4,4ʹ dichloro 2‐hydroxydiphenyl ether (DCPP)

Besides body malodour that is present on the skin itself, secretions and skin‐degradation products, skin‐borne bacteria, and malodorous substances are also transferred to clothes while wearing. Propagated by high humidity, microbial malodour production then occurs on the fabrics during wearing but also afterwards in the laundry basket. The use of mild, bleach‐free detergents and low‐temperature washing prevents the efficient removal of body soils, bacteria, and malodorous substances. Over several wearing/washing cycles, this promotes microbial propagation and malodour production on fabrics and in the washing machine (Hammer et al., 2011; Van Herreweghen et al., 2020; Riley et al., 2017). As one of the possible ways to overcome such problems, the use of antimicrobial substances formulated in the liquid laundry detergent has been proposed (Hazenkamp & Ochs, 2011; Ochs et al., 1999). 4,4ʹ dichloro 2‐hydroxydiphenyl ether (DCPP) is such an antibacterial compound (Figure 1). DCPP, available as Tinosan^®^ HP 100 (30% DCPP in 1,2‐propylene glycol), represents a non‐ionic substance that is compatible with liquid and powder detergents and exhibits broad‐spectrum antibacterial properties (Ochs et al., 1999). Applied during laundry, DCPP prevents microbial growth on washed textiles (Ochs et al., 1999). Whether—besides its antimicrobial activity—DCPP also affects microbial malodour formation on textiles is so far unknown.

The aim of this study was therefore to investigate the potential inhibitory effect of the antimicrobial DCPP on the microbial formation of IVA as a model malodorous substance in a relatively simple experimental setup. A medium adapted from James et al. (James, Cox, et al., 2013; James, Hyliands, et al., 2004) was incubated with the bacterium *Staphylococcus aureus* under various in vitro setups including shake flask cultivation and cultivation on cotton fabrics that were washed with a DCPP‐containing detergent. Physiological parameters such as growth, extracellular pH, and eventually production of malodorous IVA were followed. A GC‐FID and GC–MS method was established to analyze IVA amounts produced by *S. aureus* on fabrics.

## 2 MATERIALS AND METHODS

### 2.1 Strains and growth conditions

Strains used in this study are listed in Table 1. For growth studies, cells were grown in media adapted from James et al. (James, Cox, et al., 2013; James, Hyliands, et al., 2004). Cells were cultivated on tryptic soy agar supplemented with Tween^®^ 80 (TSAT; 30 g/L tryptone soy broth (Merck), 10 g/L yeast extract (Oxoid), 10 g/L Tween^®^ 80 (Merck), 20 g/L agar (VWR)) at 35°C for 24 h. Cells were then pre‐cultivated in 15 ml (*Corynebacterium xerosis*) or 30 ml (*Staphylococcus aureus*) tryptic soy broth supplemented with Tween^®^ 80 (TSBT; 30 g/L tryptone soy broth (Merck), 10 g/L yeast extract (Oxoid), 10 g/L Tween^®^ 80 (Merck)) at 125 rpm, 35°C for 16 h in 100 ml (*C. xerosis*) or 200 ml (*S*.* aureus*) Erlenmeyer shake flasks. Cells were harvested by centrifugation (15 min, 10,000 *g*), and the cell pellet was washed in semisynthetic medium (SSM; 0.5 g/L yeast extract (Oxoid), 3.35 g/L yeast nitrogen base (Oxoid), 0.2 g/L Tween^®^ 80 (Merck), 20 ml/L RPMI 1640 amino acids solution (Sigma‐Aldrich), 0.1 g/L L‐leucine (AppliChem), 1.6 g/L KH_2_PO_4_ (Merck), 1 g/L NaHCO_3_ (Merck), 0.38 g/L Na_2_SO_4_ (Merck), 0.1 g/L KNO_3_ (Merck), 1 g/L sodium pyruvate (AppliChem), 0.5 g/L MgCl_2_x6H_2_O (Merck), 5 g/L (NH_4_)_2_HPO_4_ (Acros Organics), 5 g/L glucose (Bernd Kraft), pH 6.0; adapted from (James, Cox, & Worrall, 2013; James, Hyliands, et al., 2004)) and centrifuged again as described above.

**TABLE 1:** Bacterial strains used in this study

Strain	References
*Staphylococcus aureus* ATCC 6538	American Type Culture Collection (ATCC)
*Corynebacterium xerosis* DSM 20170	Leibniz Institute DSMZ—German Collection of Microorganisms and Cell Cultures

For in vitro growth studies in shake flasks, the cell pellet was resuspended in SSM and absorption at 595 nm (A_595_) was determined in a photometer. Main cultures were inoculated to A_595_ = 0.1 and grown in 15 ml SSM optionally supplemented with 67 mg/L Tinosan^®^ HP 100 (BASF, corresponding to 20.1 mg/L DCPP, CAS no.: 3380‐30‐1, EC‐No. 429‐290‐0, 255 g/mol) in 100 ml Erlenmeyer shake flasks and were incubated at 35°C and 150 rpm for up to 48 h. After 0 h, 6 h, 24 h, and 48 h, aliquots were taken for A_595_ and pH measurements. For *C. xerosis*, aliquots were additionally taken after 3 h. pH of the cultures was determined using pH test strips (MColorpHast™, Merck). For *S. aureus*, after 48 h samples were taken for IVA analysis.

For growth studies on textiles, a procedure adapted from the US standard AATCC 100–2012 test method (assessment of antibacterial finishes on textile materials (AATCC, 2012)) was conducted. *S. aureus* cells were pre‐cultivated as described above, except that the pre‐cultivation was performed in 10 ml TSBT in 100 ml Erlenmeyer shake flasks.

Disks (Ø 4 cm) were punched out from treated cotton textiles and inoculated with 125 µl cell suspension (in SSM, as described above) per textile swatch. Cultivation was performed in sterile Petri dishes (Ø 5.5 cm) at 35°C under humid conditions (>90% humidity) for up to 24 h. Immediately after inoculation of the fabrics and after 24 h of incubation, cells were resuspended from the fabrics in sterile Stomacher^®^ bags 80 (Seward Ltd.) containing 10 ml buffer (1.7 g/L KH_2_PO_4_, 9.6 g/L Na_2_HPO_4_xH_2_O, 10 g/L Tween^®^ 80, 3 g/L lecithin, pH 7.4) and were agitated in Seward Stomacher^®^ 80 (Seward Ltd.) for 1 min at normal speed. Dilutions were prepared in sterile deionized water, and colony‐forming units per ml (cfu/ml) were determined on TSAT via the pour plate method after incubation of the plates at 35°C for 24 h. Cfu data presented as log_10_ values.

### 2.2 Treatment of cotton textiles

Standard white cotton fabrics (Renforcé‐1–3005, Spoerri & Co. AG, 130 g/m^2^) were used. To remove any production‐related contamination from the fabrics, they were rinsed in 90°C hot tap water for 20 min (10 g fabric/75 ml water) under agitation in a LiniTest^®^ machine (Atlas), then rinsed by hand in cold tap water for 15 s, dried and sterilized by autoclavation. 10 g of pre‐rinsed, sterile cotton textile was then laundered with 50 g of washing solution (0.487% standard European liquid laundry detergent bought in a German supermarket, optionally supplemented with 0.3% or 0.6% Tinosan^®^ HP 100 (corresponding to 0.09% or 0.18% DCPP), dissolved in tap water (water hardness: 1.75 mM calcium/magnesium ions = 9.8°dH) at room temperature (RT) in a LiniTest^®^ machine for 45 min. Rinsing of the textiles was performed with 3 x 1 L of sterile tap water at RT for 2 min. Textiles were wrung out and dried. Treatment was conducted with sterile equipment under low‐germ conditions.

### 2.3 Analysis of isovaleric acid

A gas chromatography (GC) method for the quantification of IVA was adapted from James, Cox et al. (2013). For the analysis of shake flask cultivation, cultures were harvested by centrifugation after distinct incubation periods, the supernatant was sterile‐filtered (pore size: 0.2 µm) and stored at −20°C until use. After thawing, samples were analyzed through GC using a flame ionization detector (GC‐FID). GC‐FID was performed using a 7890 gas chromatograph equipped with a split/splitless injector and FID (Agilent Technologies). Chromatographic separation was achieved using an AT‐1000 column (100% polyethylene glycol modified with nitroterephthalate; 30 m × 320 µm × 0.25 µm; Thermo Fisher Scientific) with a temperature program of 80°C for 2.5 min; 15 K/min to 155°C for 10 min. 1 µl of the sample was injected at 240°C using a split ratio of 20:1.

For the analysis of textiles, samples were stored at −20°C after specific incubation periods and before extraction. The samples were supplemented with 0.5 ml 2 M HCl/textile swatch, 0.125 ml internal standard (0.012 mg/ml hexanoic acid in ethyl acetate), and 1.125 ml ethyl acetate. Samples were gently agitated on a shaker for 2 h; reaction tubes were rotated by 90° every 30 min. After phase separation, the upper organic phase (containing IVA and the internal standard) was removed and analyzed by the means of gas chromatography‐mass spectrometry (GC–MS), since some residues from the laundry detergent on the fabrics interfered with the detection of IVA via FID. GC–MS was performed using a 7890 gas chromatograph (Agilent Technologies) coupled to a 5977A mass selective detector (MSD; Agilent Technologies) using the MassHunter software (Agilent Technologies) for instrument control and data analysis. Chromatographic separation was achieved using an AT‐1000 column (100% polyethylene glycol modified with nitroterephthalate; 30 m × 320 µm × 0.25 µm; Thermo Fisher Scientific), with a temperature program of 80°C for 2.5 min; 15 K/min to 155°C; and 155°C for 30 min. 1 µl of the sample was injected at 250°C using a split ratio of 20:1. The MSD was operated in selected ion‐monitoring mode acquiring m/z 60, 87, and 43 for IVA and *m*/*z* 60, 73, and 87 for hexanoic acid (internal standard). Successful extraction of IVA from textiles was confirmed by analysis of a water‐treated textile that was abiotically incubated with IVA.

Recovery rates for IVA were ≥90%.

### 2.4 Statistical analysis

Data are presented as arithmetic mean values and standard deviations of 3 independent biological replicates, if not stated differently. Statistical analyses were carried out as a one‐way analysis of variance (ANOVA). If significant differences were found, ANOVA was followed by Tukey's honestly significant difference (HSD) test, results presented as *p* values (*p*). Analyses were performed assuming independent data and standard weighted means. For statistical analysis of cfu data, Student's *t*‐test was performed for paired values with equal variances. The significance level *α* = 0.05 was chosen.

## 3 RESULTS

### 3.1 DCPP inhibits the formation of the VFA isovaleric acid by *S. aureus* in a semisynthetic medium

In the body malodour formation, staphylococci represent key microorganisms for the production of IVA in human sweat. In a semisynthetic medium that functionally mimics human sweat, SSM (adapted from James, Cox et al. (2013)), the growth of *S. aureus* was followed in shake flasks as a sweat model system for up to 48 h in the presence or absence of the antimicrobial DCPP.

**FIGURE 2:**
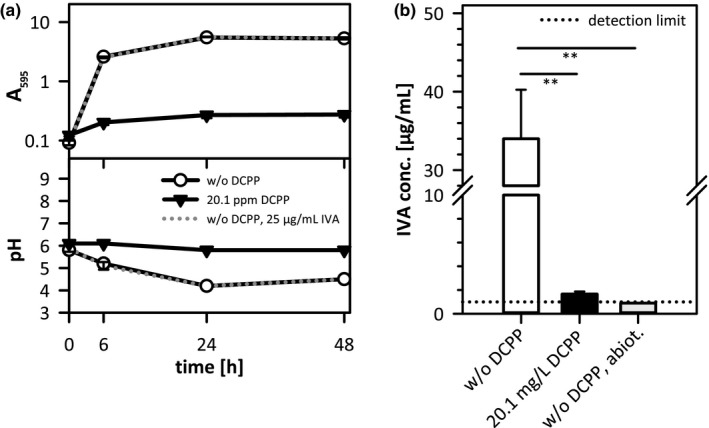
Characterization of growth and extracellular IVA concentrations of *Staphylococcus aureus* in SSM supplemented with or w/o 20.1 mg/L DCPP or 25 µg/ml IVA. (a) Upper panel: cell density displayed as A_595_ values, lower panel: extracellular pH. (b) Corresponding extracellular IVA concentrations after 48 h cultivation of *S. aureus*; dotted line: detection limit = 1 µg/ml IVA. Statistical analysis revealed *p* < 0.01 (**) for the sample w/o DCPP versus the sample with 20.1 mg/L DCPP and the abiotic sample w/o DCPP. Bacteria were grown aerobically at 35°C and 150 rpm. Three replicates each.

A relatively high concentration of 20.1 mg/L DCPP was chosen to demonstrate the general principles of the processes involved. 20.1 mg/L DCPP strongly impaired the growth of *S. aureus*, reaching 20 times lower maximum cell densities than without DCPP (A_595_ of around 0.275 and 5.3, respectively; Figure 2a). This highlights the bacteriostatic mode of action of the substance that does not enable the killing of the bacteria but rather inhibits microbial growth. The pH of the sample w/o DCPP dropped from  6 to approx.   4 and subsequently mildly increased again. In contrast, the sample containing DCPP revealed only a minor pH drop. Furthermore, the impact of IVA on the growth of *S. aureus* was followed. Cell densities and pH profiles were almost identical to the sample w/o IVA, proving that the potential production of the substance did not affect the growth of the bacterium.

After 48 h cultivation, samples were analyzed regarding their IVA contents (Figure 2b). Therefore, a GC‐FID‐based analysis method was established. In the absence of DCPP, *S. aureus* produced 34 µg/ml ±6.2 µg/ml IVA. In contrast, with DCPP only 1.7 µg/ml ±0.2 µg/ml IVA were formed, and bacterial growth was significantly impaired. After 24 h of cultivation, IVA concentrations were in a similar range as after 48 h (24 h: 102.1% ±1.1% and 86.0% ±0.9% of the corresponding 48 h‐amounts with or w/o DCPP, respectively; pooled data from two biological replicates with 2 technical replicates for IVA analysis each; not shown in the figure). IVA was not abiotically produced (IVA content below the detection limit), proving that the entire amount of the malodorous substance resulted from the metabolic activity of *S. aureus*. These data emphasize our assumption that DCPP as an antimicrobial substance is capable of inhibiting growth and IVA production of a malodour‐associated bacterium in an artificial sweat model.

**FIGURE 3:**
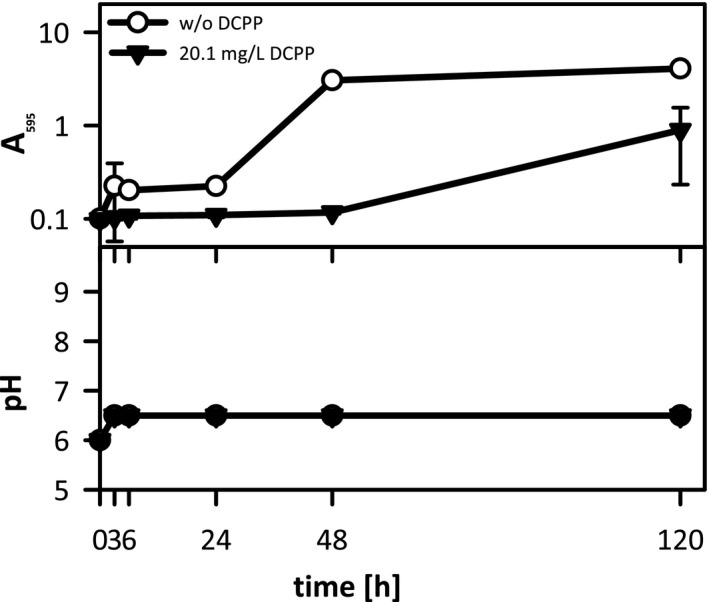
Characterization of *Corynebacterium xerosis* growth in SSM supplemented with or w/o 20.1 mg/L DCPP. Upper panel: cell density displayed as *A*_595_ values, lower panel: extracellular pH. Bacteria were grown aerobically at 35°C and 150 rpm. Three replicates each; for sample w/o DCPP at 48 h (*A*_595_), only 2 out of 3 replicates were evaluated.

The growth of *C. xerosis* in SSM supplemented with 20.1 mg/L DCPP was impaired as well (Figure 3), however, to a lesser extent than that observed for *S. aureus*. Maximum cell densities of the DCPP‐containing sample were approx. *A*_595_ = 0.9 after 120 h cultivation, in contrast to the sample w/o DCPP, where *C. xerosis* reached *A*_595_ of 4.1. Unlike *S. aureus*, the pH profile did not vary tremendously during cultivation and was not affected by DCPP. These data highlight that, besides its inhibiting effects on staphylococcal growth and IVA formation, DCPP also bears the antimicrobial activity against corynebacteria, the second important class of malodour‐producing bacteria.

### 3.2 Inhibition of isovaleric acid formation in *S. aureus* on DCPP‐treated textiles

**FIGURE 4:**
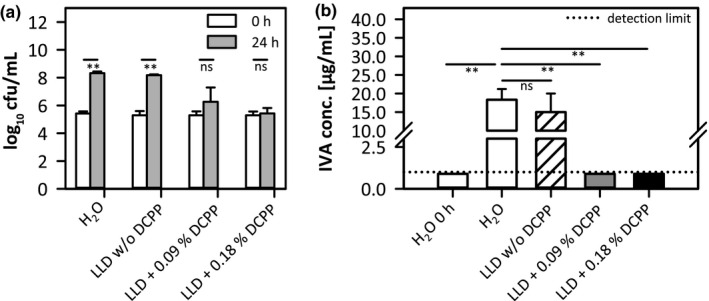
Characterization of growth and extracellular IVA concentrations of *S. aureus* in SSM on cotton fabrics treated with water (H_2_O), a standard LLD supplemented with or w/o 0.09% or 0.18% DCPP. (a) Cell density displayed as log_10_ cfu/ml values, determined directly after inoculation (0 h) or after 24 h incubation (24 h). Statistical analysis revealed *p* < 0.01 (**) for the H_2_O‐treated sample and the LLD‐treated sample w/o DCPP (0 h vs. 24 h). Samples containing DCPP revealed *p* > 0.05. (b) Corresponding extracellular IVA concentrations after 24 h incubation of the fabrics with *S. aureus*; dotted line: detection limit = 1 µg/ml IVA. Textile samples incubated for 24 h, except for sample H_2_O 0 h, representing the reference IVA level at test start. Statistical analysis revealed *p* < 0.01 (**) for H_2_O‐treated sample incubated for 24 h versus the same sample tested immediately after inoculation (0 h), as well as versus samples treated with LLD +0.09% or 0.18% DCPP. The sample treated with LLD w/o DCPP revealed *p* > 0.05 (nonsignificant, ns). Bacteria were grown aerobically under humid conditions at 35°C. Three replicates each.

In addition to the shake flask model, a different test system based on fabrics was established to investigate the effect of DCPP on IVA production in a laundry setting. 0.09% or 0.18% DCPP was added to a commercially available liquid laundry detergent (LLD). Cotton fabrics that were washed with the test detergent were inoculated with *S*.* aureus* in SSM as described above. Cotton represents a suitable material for our studies as *Staphylococcus* species tend to grow better on cotton than on polyester (Callewaert, de Maeseneire, et al., 2014). Moreover, Munk et al. demonstrated that odor generated on cotton during wet storage was significantly greater than on polyester (Munk et al., 2001). This may be due to greater water absorbency of the hydrophilic and porous cellulosic cotton fibers. A stronger odor that is sometimes encountered for polyester fibers might be caused by organic odorous substances that tend to stick stronger to polyester than to cotton and thus are more difficult to remove in washing. (Munk et al., 2001). This phenomenon might be of importance for the field of malodour in clothes but is not in the scope of this study. The growth of the bacterium on the textiles was determined directly after inoculation and after 24 h of incubation (Figure 4a). For the fabrics treated with either water or LLD w/o DCPP, significant growth of *S. aureus* could be observed over 24 h of incubation (from 5.4 log_10 _cfu/ml immediately after inoculation to 8.2 log_10 _cfu/ml after 24 h), demonstrating that potential detergent residues present on the fabrics did not exhibit antimicrobial activity under the test conditions applied. In contrast, on fabrics treated with LLD containing DCPP, no significant growth occurred during 24 h incubation.

Extraction and GC‐MS analysis of IVA that was produced on the fabrics during staphylococcal growth revealed significant microbial IVA production on water‐treated and LLD w/o DCPP‐treated textiles (18.3 ± 2.9 µg/ml) during 24 h (Figure 4b).

IVA could not be detected on textiles treated with LLD containing DCPP, which is consistent with the finding that these fabrics revealed strong growth inhibition of *S. aureus*. This outlines the antimicrobial effect of DCPP deposited on the fabrics during laundry that impairs *S. aureus*’ growth and simultaneously prevents the microbial formation of malodorous IVA in a sweat model system on fabrics.

## 4 DISCUSSION

Body malodour is a complex phenomenon comprising several types of sweat glands producing various odorless body secretions that are being metabolized by a consortium of skin‐resident microorganisms to a wide variety of malodorous compounds. Fabrics with direct skin contact are prone to malodour formation due to the transfer of body soils and microorganisms from the skin to the textile. Textile malodour may be caused either by direct transfer of malodorous compounds from the skin or by microbial transformation of odorless body soils to malodorous substances on the fabrics. A laundry process providing effective removal of body soils and microorganisms is of crucial importance to manage malodour control on textiles. While bleach‐containing powder detergents and high‐temperature laundry are very effective in the elimination of organic stains and microorganisms, washing at low temperatures with mild, bleach‐free detergents often results in significant microbial residues and incomplete removal of organic stains on washed fabrics. Analysis of bacterial metabolism of the amino acid L‐leucine that is present in human sweat to the typical malodorous substance IVA represents a quantitative test method that can be used for investigation of malodour‐reducing technologies and their mode of action.

This study is, to our knowledge, the first one demonstrating the inhibition of the microbial production of IVA, one of the key body malodour substances (Caroprese et al., 2009; James, Austin et al., 2013; James, Cox et al., 2013; James, Hyliands et al., 2004; Kanda et al., 1990; Leyden et al., 1981), by an antimicrobial, DCPP, deposited on fabrics.

SSM, a growth medium adapted from James et al. (James, Cox et al., 2013) mimicking human sweat and containing L‐leucine, which is the substrate for IVA synthesis, was used for cultivation studies. In SSM, 20.1 mg/L DCPP strongly impaired the growth of *S. aureus*, a representative of the genera of staphylococci. Staphylococci are known as key skin‐resident bacteria involved in body malodour formation (Bawdon et al., 2015; James, Cox et al., 2013; James, Hyliands et al., 2004). On clothes, predominantly Gram‐positive bacteria such as *Staphylococcus epidermidis* and *Staphylococcus hominis* and corynebacteria were observed (Callewaert, de Maeseneire et al., 2014). In this study, *S. aureus* ATCC 6538 was used. This organism might not be the ideal representative of body malodour‐producing staphylococci. However, it is a common organism found in many international microbiological standard test methods and was used as a representative Gram‐positive bacterium able to metabolize branched aliphatic amino acids, such as L‐leucine, to volatile degradation products, like IVA (James Cox, & Worrall, 2013; James, Hyliands et al., 2004). For this model study, the relative abundance of a certain bacterial species is not of key importance, but rather its ability to perform these metabolic pathways. SSM was shown to be suitable for the cultivation of body malodour‐producing staphylococci and the analysis of the resulting malodorous substances. Hence, SSM functionally mimics but does not fully replace human sweat. In contrast to SSM, the most prominent artificial sweat compositions found in the scientific literature like the British standard BS EN 1811:2001+A1:2015 (reference test method for release of nickel from all post‐assemblies which are inserted into pierced parts of the human body and articles intended to come into direct and prolonged contact with the skin (BIS, 2015)), the European standard EN ISO 105‐E04:2013 (textiles—tests for color fastness—part E04: color fastness to perspiration (DIN, 2013)) and the US standard AATCC 15‐2013 (colorfastness to perspiration (AATCC, 2013) compositions or SCIN (Callewaert, Buysschaert et al., 2014) contain considerable amounts of sodium chloride, lactic acid and urea but no protein/peptides, as summarized by Kulthong or Callewaert (Callewaert, Buysschaert et al., 2014; Kulthong et al., 2010).

Under these conditions, we observed bacterial growth inhibition rather than bactericidal activity (Figure 2a). DCPP’s bacteriostatic activity was further confirmed by a very mild and delayed pH drop in the DCPP‐containing sample that reflected the impaired metabolism. In contrast, a characteristic pH drop in the sample w/o DCPP revealed active metabolism and formation of fermentation products (Somerville et al., 2002). Surprisingly, little is known about the antimicrobial mechanism of DCPP. While DCPP provides bacteriostatic activity at low concentrations as found in typical in‐use dilutions of LLDs (Figures 2 and 3), it exhibits rapid bactericidal activity at higher concentrations (Ochs et al., 1999). As observed for other phenolic compounds including diphenylethers, it can be assumed that the lipophilic DCPP exerts perturbing effects on bacterial membranes. This results in impacts on membrane functionality, and, esp. at higher concentrations, on membrane integrity (McDonnell & Russel, 1999; Villalaín et al., 2001). Furthermore, an inhibitory effect of DCPP on membrane lipid synthesis could be concluded from studies on DCPP complexes with the enoyl‐acyl carrier protein reductase of *Helicobacter pylori* (Lee et al., 2007). Furthermore, the bacteriostatic effect of DCPP is accompanied by a reduction of the metabolization of L‐leucine to IVA, caused by a lower number of bacteria and indicated by the finding that almost no IVA was produced in the DCPP‐containing sample, while significant amounts were found in the absence of DCPP (Figure 2b). Additionally, we could show the broad‐spectrum antimicrobial activity of DCPP in medium containing typical components of human sweat against the second important class of body malodour‐producing microorganisms: corynebacteria (Figure 3; Callewaert et al., 2013; Leyden et al., 1981; Shehadeh & Kligman, 1963)). Further studies are required to provide more evidence for this assumption. DCPP exhibited a lower impact on the growth of *C. xerosis* than observed for *S. aureus*. This observation is in consistence with previous results on minimal inhibitory concentration (MIC) values for DCPP, revealing a lower susceptibility of *C. xerosis* (MIC = 6 mg/L) compared to *S. aureus* and other skin‐borne staphylococci, such as *S. epidermidis* (MIC = 0.06 mg/L) (Ochs et al., 1999). These findings clearly outline that our simplified experimental malodour setup is suitable for detecting the effectiveness of an antimicrobial, here DCPP, in the inhibition of one of the major body malodour producers, *Staphylococcus*. Furthermore, the data indicate that corynebacterial malodour production might also be affected.

We wondered whether these effects could be confirmed under laundry care conditions. Cotton fabrics washed with a standard LLD containing 0.09% to 0.18% DCPP, a realistic concentration used in laundry care, were inoculated with a suspension of *S*.* aureus* and SMM (Figure 4). Confirming the data obtained in shake flask cultivation, treatment with a DCPP‐containing detergent inhibited staphylococcal growth and IVA formation on fabrics. The data demonstrate that DCPP deposited on fabrics during laundry effectively inhibits staphylococcal IVA formation.

During laundry with DCPP‐containing detergents, small amounts of DCPP deposit on fabrics. This effect might be due to DCPP’s hydrophobic nature; the water/fabric partition coefficient appears to be such that the deposition on the fabric occurs. In this study, the levels of DCPP deposition were not determined. In a study on DCPP‐containing softeners, where similar application rates of DCPP per kg of textile were applied, approx. 2.5 mg DCPP per kg of textile were found (Metcalfe et al., 2013). It should be noted that a concentration in mg/kg textile cannot be compared with a concentration in mg/L in a solution. On the fabric, the local concentration of DCPP in the liquid water present in the pores of the cotton represents the relevant biocidal concentration. This latter concentration is not easily and accurately determinable. The fact that significant inhibition of *S. aureus’* growth on textile was observed shows that this local effective concentration lies at least in the range of MIC value for *S. aureus* (Ochs et al., 1999). In the liquid culture experiment, the concentration of 20.1 mg/L is clearly above the MIC value and was chosen to demonstrate general principles of malodour prevention by DCPP.

To tackle increased microbial contamination of fabrics (Hammer et al., 2011; Riley et al., 2017) and emerging malodour formation caused by mild laundry processes, several technologies are available, as previously reviewed (Hazenkamp & Ochs, 2011). These technologies, however, have several limitations for modern liquid laundry detergents. Bleach‐based systems like sodium hypochlorite or peroxides are characterized by high oxidative power and/or chemical instability. These substances exhibit immediate disinfection but can destroy enzymes and perfumes in the liquid detergent and decompose under the formation of oxygen gas. Sodium hypochlorite and hydrogen peroxide are only available in separate laundry adjuncts and can indeed be of value in microbial control. They might, however, also damage dyes on colored clothes. A combination of solid sodium percarbonate and tetraacetylethylenediamine (TAED) that forms biocidal peracetic acid in situ during washing is only applicable in laundry powder detergents. Quaternary ammonium compounds (quats) like benzalkonium chloride or didecyldimethylammonium chloride are powerful biocides, which may also show a certain remnant antimicrobial effect on textiles. Due to their cationic nature, they are incompatible with laundry detergents, which are mostly based on anionic surfactants. Quats can be used in separate hygienic after‐rinse products and be of value to control bacteria on textiles, but they are not applicable in the main wash. Silver ions are used in some antimicrobial textiles (Openshaw et al., 2016) but precipitate in alkaline liquid detergents. Likewise, other classes of biocides cannot be used in liquid laundry detergents: Aldehydes (glutaraldehyde, formaldehyde) are unstable in alkaline liquids and would react with enzymes during storage. Alcohols are not effective in high dilution. Hence, DCPP exhibiting antibacterial and malodour‐reducing effects on washed textiles represents an interesting antimicrobial technology for improved hygiene with bleach‐free detergents that are intended to be used especially for low‐temperature laundry. Concentrations between 0.09% and 0.18% DCPP are effective in an LLD under standard laundry conditions and show strong preventive effects on the formation of malodorous IVA under experimental conditions.

**FIGURE 5:**
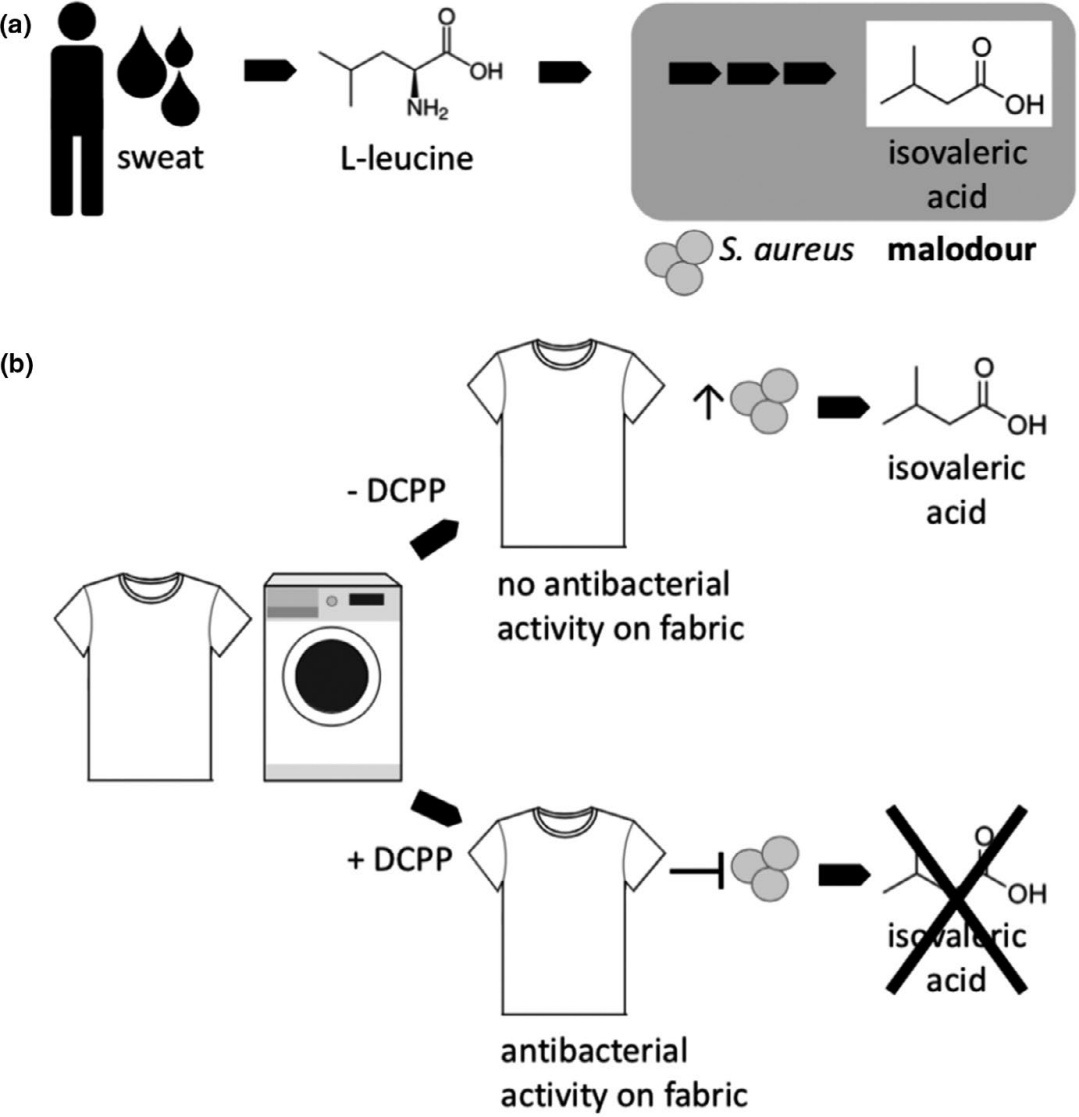
Model of microbial formation of IVA and inhibition of IVA formation by DCPP. (a) L‐leucine present in human sweat is degraded by staphylococci, for example, *S. aureus*, to the malodorous substance IVA (adapted from James, Cox, et al. (2013))). (b) Treatment of fabrics with a detergent supplemented with (+) or without (−) DCPP; in the absence of DCPP, microbial growth and production of IVA occur on fabrics, fabric treatment with a DCPP‐containing detergent leads to DCPP deposition and prevention of microbial growth and IVA production on fabrics.

The simplified experimental setup of this study is not considered to reflect realistic conditions where a large number and strong variation in microorganisms, biotransformations, and odorous substances can be found. This in vitro study merely represents a model study that demonstrates how an antimicrobial substance may inhibit bacterial transformations, which can lead to reduced malodour on clothes. The processes demonstrated in this study are summarized in a model in Figure 5: By washing fabrics with a DCPP‐containing detergent, ppm levels of the antimicrobial substance are deposited on the textiles. If body soil, sweat, and skin‐borne staphylococci are transferred to the fabrics during wearing, the microbial formation of malodorous IVA from L‐leucine (Figure 5a) is effectively inhibited by the antimicrobial DCPP, while in the absence of DCPP microbial growth and metabolism and eventually IVA formation on fabrics are promoted in vitro (Figure 5b).

In vivo studies with fabrics that were washed with a DCPP‐containing detergent and which were worn by volunteers could shed more light on the relevance of these findings and might be the subject for future studies. Although biocides, unlike antibiotics, have a non‐specific mode of action, the use of broad‐spectrum biocides might impact the composition of microbial communities, as recently shown (Callewaert et al., 2020). Therefore, the effect of DCPP on the microbiome and consequences of potential alterations thereof for malodour production remain interesting questions which may be addressed in future in vivo studies, as well. For all uses of biocides in consumer care, besides general toxicological and environmental hazards, the risk assessment for the products need to consider specific features of biocidal actives, such as resistance and cross‐resistance development to other biocides or antibiotics (e.g., (Condell et al., 2012; Edgar & Bibi, 1997; Tandukar et al., 2013)), and their relevance under realistic use conditions (SCENIHR, 2009, Bloomfield, 2002, Smith et al., 2012).

## 5 CONCLUSIONS

In this study, we have established GC‐FID‐ and GC‐MS‐based methods to analyze microbially produced IVA in artificial human sweat‐mimicking medium and on fabrics. We demonstrated that IVA is produced in this medium on fabrics by the skin‐resident bacterium *S. aureus*. Moreover, we show a relatively simple experimental setup for analyzing the effects of an antimicrobial on body malodour. The study provides evidence that staphylococcal growth, metabolism, and IVA production in a medium that functionally mimics sweat and on fabrics contaminated with the bacterium can be inhibited by an antimicrobial substance, DCPP, that is deposited on fabrics during laundry with a DCPP‐containing detergent. Furthermore, the activity of the antimicrobial against corynebacteria, important body malodour producers, was shown, suggesting a broad‐spectrum malodour inhibition on fabrics. Thus, antimicrobial technologies showing deposition on textiles during the laundry process represent an interesting approach to increase hygiene in laundry care where low‐temperature washing with mild detergents elevates the problem of microbial contamination and malodour formation.

## CONFLICT OF INTEREST

All authors are employees of BASF. BASF declares a commercial interest regarding the manufacturing and marketing of 4,4ʹ dichloro 2‐hydroxydiphenyl ether.

## AUTHOR CONTRIBUTIONS

**Sonja Mayer:** Conceptualization (supporting); Formal analysis (lead); Methodology (equal); Validation (lead); Visualization (lead); Writing‐original draft (lead); Writing‐review & editing (lead). **Menno Hazenkamp:** Conceptualization (lead); Funding acquisition (lead); Methodology (equal); Project administration (lead); Supervision (lead); Writing‐review & editing (supporting). **Martin Kluttig:** Methodology (equal); Writing‐review & editing (supporting). **Dietmar Ochs:** Funding acquisition (lead); Supervision (supporting); Writing‐review & editing (supporting).

## ETHICS STATEMENT

None required.

## Data Availability

The datasets generated and/or analyzed during the current study are provided in full in this publication.

## References

[mbo31174-bib-0001] AATCC, A. A. O. T. C. A. C . (2012). AATCC test method 100‐2012: Antibacterial Finishes on Textile Materials: Assessment of. AATCC Technical Manual. Research Triangle Park, NC 27709 USA.

[mbo31174-bib-0002] AATCC, A. A. O. T. C. A. C . (2013). AATCC test method 15‐2013: Colorfastness to Perspiration. AATCC Technical Manual. Research Triangle Park, NC 27709 USA.

[mbo31174-bib-0003] Austin, C., & Ellis, J. (2003). Microbial pathways leading to steroidal malodour in the axilla. Journal of Steroid Biochemistry and Molecular Biology, 87, 105–110. 10.1016/s0960-0760(03)00387-x 14630096

[mbo31174-bib-0004] Bawdon, D., Cox, D. S., Ashford, D., James, A. G., & Thomas, G. H. (2015). Identification of axillary Staphylococcus sp. involved in the production of the malodorous thioalcohol 3‐methyl‐3‐sufanylhexan‐1‐ol. FEMS Microbiology Letters, 362, 1–10. 10.1093/femsle/fnv111 26163522

[mbo31174-bib-0005] BSI, B. S. I. (2015). BS EN 1811:2011+A1:2015 Reference test method for release of nickel from all post assemblies which are inserted into pierced parts of the human body and articles intended to come into direct and prolonged contact with the skin. United Kingdom.

[mbo31174-bib-0006] Bloomfield, S. F. (2002). Significance of biocide usage and antimicrobial resistance in domiciliary environments. Journal of Applied Microbiology, 92, 144S–157S. 12000623

[mbo31174-bib-0007] Callewaert, C., Buysschaert, B., Vossen, E., Fievez, V., van de Wiele, T., & Boon, N. (2014). Artificial sweat composition to grow and sustain a mixed human axillary microbiome. Journal of Microbiol Methods, 103, 6–8. 10.1016/j.mimet.2014.05.005 24858451

[mbo31174-bib-0008] Callewaert, C., de Maeseneire, E., Kerckhof, F. M., Verliefde, A., van de Wiele, T., & Boon, N. (2014). Microbial odor profile of polyester and cotton clothes after a fitness session. Applied and Environment Microbiology, 80, 6611–6619. 10.1128/AEM.01422-14 PMC4249026 25128346

[mbo31174-bib-0009] Callewaert, C., Kerckhof, F. M., Granitsiotis, M. S., van Gele, M., van de Wiele, T., & Boon, N. (2013). Characterization of Staphylococcus and Corynebacterium clusters in the human axillary region. PLoS One, 8, e70538. 23950955 10.1371/journal.pone.0070538 PMC3741381

[mbo31174-bib-0010] Callewaert, C., Ravard Helffer, K., & Lebaron, P. (2020). Skin microbiome and its interplay with the environment. American Journal of Clinical Dermatology, 21, 4–11. 32910439 10.1007/s40257-020-00551-x PMC7584520

[mbo31174-bib-0011] Caroprese, A., Gabbanini, S., Beltramini, C., Lucchi, E., & Valgimigli, L. (2009). HS‐SPME‐GC‐MS analysis of body odor to test the efficacy of foot deodorant formulations. Skin Research and Technology, 15, 503–510. 19832965 10.1111/j.1600-0846.2009.00399.x

[mbo31174-bib-0012] Condell, O., Iversen, C., Cooney, S., Power, K. A., Walsh, C., Burgess, C., & Fanning, S. (2012). Efficacy of biocides used in the modern food industry to control *Salmonella enterica*, and links between biocide tolerance and resistance to clinically relevant antimicrobial compounds. Applied and Environment Microbiology, 78, 3087–3097. 10.1128/AEM.07534-11 PMC3346496 22367085

[mbo31174-bib-0013] Costello, E. K., Lauber, C. L., Hamady, M., Fierer, N., Gordon, J. I., & Knight, R. (2009). Bacterial community variation in human body habitats across space and time. Science, 326, 1694–1697. 19892944 10.1126/science.1177486 PMC3602444

[mbo31174-bib-0014] DIN, D. I. F. N. (2013). EN ISO 105‐E04:2013: Textiles ‐ Tests for colour fastness ‐ part E04: Colour fastness to perspiration. Berlin, Germany.

[mbo31174-bib-0015] Edgar, R., & Bibi, E. (1997). MdfA, an *Escherichia coli* multidrug resistance protein with an extraordinarily broad spectrum of drug recognition. Journal of Bacteriology, 179, 2274–2280. 9079913 10.1128/jb.179.7.2274-2280.1997 PMC178964

[mbo31174-bib-0016] Gower, D. B., Holland, K. T., Mallet, A. I., Rennie, P. J., & Watkins, W. J. (1994). Comparison of 16‐androstene steroid concentrations in sterile apocrine sweat and axillary secretions: interconversions of 16‐androstenes by the axillary microflora–a mechanism for axillary odour production in man? Journal of Steroid Biochemistry and Molecular Biology, 48, 409–418. 10.1016/0960-0760(94)90082-5 8142319

[mbo31174-bib-0017] Hammer, T. R., Mucha, H., & Hoefer, D. (2011). Infection risk by dermatophytes during storage and after domestic laundry and their temperature‐dependent inactivation. Mycopathologia, 171, 43–49. 20652833 10.1007/s11046-010-9347-9

[mbo31174-bib-0018] Hasegawa, Y., Yabuki, M., & Matsukane, M. (2004). Identification of new odoriferous compounds in human axillary sweat. Chemistry & Biodiversity, 1, 2042–2050. 17191839 10.1002/cbdv.200490157

[mbo31174-bib-0019] Hazenkamp, M., & Ochs, D. (2011). An antimicrobial shield for textiles washed in cold water. HPC Today, 6(2), 20–23.

[mbo31174-bib-0020] Holland, K. T. (1993). Nutrition of cutaneous resident microorganisms, The Skin Microflora and Microbial Skin Disease. Cambridge University Press.

[mbo31174-bib-0021] Holland, K. T., Gribbon, E. M., & Marshall, J. (1990). Qualitative and quantitiative assay for detection of callus degrading activity by bacteria. Letters in Applied Microbiology, 224–227.

[mbo31174-bib-0022] Holland, K. T., Marshall, J., & Taylor, D. (1992). The effect of dilution rate and pH on biomass and proteinase production by Micrococcus sedentarius grown in continuous culture. Journal of Applied Bacteriology, 72, 429–434. 10.1111/j.1365-2672.1992.tb01856.x 1618719

[mbo31174-bib-0023] Huang, C. T., Chen, M. L., Huang, L. L., & Mao, I. F. (2002). Uric acid and urea in human sweat. Chinese Journal of Physiology, 109–115. 12817713

[mbo31174-bib-0024] James, A. G., Austin, C. J., Cox, D. S., Taylor, D., & Calvert, R. (2013). Microbiological and biochemical origins of human axillary odour. FEMS Microbiology Ecology, 83, 527–540. 23278215 10.1111/1574-6941.12054

[mbo31174-bib-0025] James, A. G., Casey, J., Hyliands, D., & Mycock, G. (2004). Fatty acid metabolism by cutaneous bacteria and its role in axillary malodour. World Journal of Microbiology & Biotechnology, 20, 787–793.

[mbo31174-bib-0026] James, A. G., Cox, D., & Worrall, K. (2013). Microbiological and biochemical origins of human foot malodour. Flavour and Fragrance Journal, 28, 231–237.

[mbo31174-bib-0027] James, A. G., Hyliands, D., & Johnston, H. (2004). Generation of volatile fatty acids by axillary bacteria. International Journal of Cosmetic Science, 26, 149–156. 18494871 10.1111/j.1467-2494.2004.00214.x

[mbo31174-bib-0028] Kanda, F., Yagi, E., Fukuda, M., Nakajima, K., Ohta, T., & Nakata, O. (1990). Elucidation of chemical compounds responsible for foot malodour. British Journal of Dermatology, 122, 771–776. 10.1111/j.1365-2133.1990.tb06265.x 2369557

[mbo31174-bib-0029] Kulthong, K., Srisung, S., Boonpavanitchakul, K., Kangwansupamonkon, W., & Maniratanachote, R. (2010). Determination of silver nanoparticle release from antibacterial fabrics into artificial sweat. Part Fibre Toxicol, 7, 8. 20359338 10.1186/1743-8977-7-8 PMC2861638

[mbo31174-bib-0030] Lee, H. H., Moon, J., & Suh, S. W. (2007). Crystal structure of the Helicobacter pylori enoyl‐acyl carrier protein reductase in complex with hydroxydiphenyl ether compounds, triclosan and diclosan. Proteins, 69, 691–694. 10.1002/prot.21586 17879346

[mbo31174-bib-0031] Leyden, J. J., McGinley, K. J., Holzle, E., Labows, J. N., & Kligman, A. M. (1981). The microbiology of the human axilla and its relationship to axillary odor. The Journal of Investigative Dermatology, 77, 413–416. 7288207 10.1111/1523-1747.ep12494624

[mbo31174-bib-0032] Longshaw, C. M., Wright, J. D., Farrell, A. M., & Holland, K. T. (2002). Kytococcus sedentarius, the organism associated with pitted keratolysis, produces two keratin‐degrading enzymes. Journal of Applied Microbiology, 93, 810–816. 12392527 10.1046/j.1365-2672.2002.01742.x

[mbo31174-bib-0033] Marshall, J., Holland, K. T., & Gribbon, E. M. (1988). A comparative study of the cutaneous microflora of normal feet with low and high levels of odour. Journal of Applied Bacteriology, 65, 61–68. 10.1111/j.1365-2672.1988.tb04318.x 3145263

[mbo31174-bib-0034] McDonnell, G., & Russel, D. (1999). Antiseptics and disinfectants: activity, action, and resistance. Clinical Microbiology Reviews, 12, 147–179. 9880479 10.1128/cmr.12.1.147 PMC88911

[mbo31174-bib-0035] Metcalfe, K., Smith, I. K., & Theobald, A. J. (2013). Improvements relating to fabric conditioners. PCT/EP2013/057512. London, UK: Unilever Plc.

[mbo31174-bib-0036] Minhas, G. S., Bawdon, D., Herman, R., Rudden, M., Stone, A. P., James, A. G., Thomas, G. H., & Newstead, S. (2018). Structural basis of malodour precursor transport in the human axilla. Elife, 7, e34995. 29966586 10.7554/eLife.34995 PMC6059767

[mbo31174-bib-0037] Munk, S., Johansen, C., Stahnke, L. H., & Adler‐Nissen, J. (2001). Microbial survival and odor in laundry. Journal of Surfactants and Detergents, 4, 385–394.

[mbo31174-bib-0038] Natsch, A., Derrer, S., Flachsmann, F., & Schmid, J. (2006). A broad diversity of volatile carboxylic acids, released by a bacterial aminoacylase from axilla secretions, as candidate molecules for the determination of human‐body odor type. Chemistry & Biodiversity, 3, 1–20. 10.1002/cbdv.200690015 17193210

[mbo31174-bib-0039] Natsch, A., Gfeller, H., Gygax, P., Schmid, J., & Acuna, G. (2003). A specific bacterial aminoacylase cleaves odorant precursors secreted in the human axilla. Journal of Biological Chemistry, 278, 5718–5727. 10.1074/jbc.M210142200 12468539

[mbo31174-bib-0040] Natsch, A., Schmid, J., & Flachsmann, F. (2004). Identification of odoriferous sulfanylalkanols in human axilla secretions and their formation through cleavage of cysteine precursors by a C‐S Lyase isolated from axilla bacteria. Chemistry & Biodiversity, 1, 1058–1072. 10.1002/cbdv.200490079 17191898

[mbo31174-bib-0041] Nordstrom, K. M., McGinley, K. J., Cappiello, L., Zechman, J. M., & Leyden, J. J. (1987). Pitted keratolysis. The role of Micrococcus sedentarius. Archives of Dermatology, 123, 1320–1325. 3310909 10.1001/archderm.123.10.1320

[mbo31174-bib-0042] Ochs, D., Hoffstetter, F., & Schnyder, M. (1999). A new antimicrobial active for household products. SOFW Journal, 11, 1–7.

[mbo31174-bib-0043] Openshaw, J. J., Morris, W. M., Lowry, G. V., & Nazmi, A. (2016). Reduction in bacterial contamination of hospital textiles by a novel silver‐based laundry treatment. American Journal of Infection Control, 44, 1705–1708. 27561434 10.1016/j.ajic.2016.06.021

[mbo31174-bib-0044] Riley, K., Williams, J., Owen, L., Shen, J., Davies, A., & Laird, K. (2017). The effect of low‐temperature laundering and detergents on the survival of *Escherichia coli* and *Staphylococcus aureus* on textiles used in healthcare uniforms. Journal of Applied Microbiology, 123, 280–286. 28489297 10.1111/jam.13485

[mbo31174-bib-0045] SCENIHR, S. C. O. E. A. N. I. H. R. (2009). Assessment of the antibiotic resistance effects of biocides. European Commission. https://ec.europa.eu/health/ph_risk/committees/04_scenihr/docs/scenihr_o_021.pdf

[mbo31174-bib-0046] Shehadeh, N., & Kligman, A. M. (1963). The bacteria responsible for axillary Odor. Ii. Journal of Investigative Dermatology, 41, 39–43. 14043010

[mbo31174-bib-0047] Shelley, W. B., Hurley, H. J., & Nichols, A. C.. (1953). Axillary odor; experimental study of the role of bacteria, apocrine sweat, and deodorants. AMA Archives of Dermatology and Syphilology, 68, 430–446. 13091383 10.1001/archderm.1953.01540100070012

[mbo31174-bib-0048] Smith, R. S., Bloomfield, S. F., & Rook, G. A. (2012). The Hygiene hypothesis and its implications for home hygiene, lifestyle and public health. International Scientific Forum on Home Hygiene. https://www.ifh‐homehygiene.org/system/files_force/publications/Hygiene%20hypothesis%20review_19092012.pdf

[mbo31174-bib-0049] Somerville, G. A., Chaussee, M. S., Morgan, C. I., Fitzgerald, J. R., Dorward, D. W., Reitzer, L. J., & Musser, J. M. (2002). Staphylococcus aureus aconitase inactivation unexpectedly inhibits post‐exponential‐phase growth and enhances stationary‐phase survival. Infection and Immunity, 70, 6373–6382. 12379717 10.1128/IAI.70.11.6373-6382.2002 PMC130419

[mbo31174-bib-0050] Tandukar, M., Oh, S., Tezel, U., Konstantinidis, K. T., & Pavlostathis, S. G. (2013). Long‐term exposure to benzalkonium chloride disinfectants results in change of microbial community structure and increased antimicrobial resistance. Environmental Science and Technology, 47, 9730–9738. 23924280 10.1021/es401507k

[mbo31174-bib-0051] Taylor, D., Daulby, A., Grimshaw, S., James, G., Mercer, J., & Vaziri, S. (2003). Characterization of the microflora of the human axilla. International Journal of Cosmetic Science, 25, 137–145. 18494895 10.1046/j.1467-2494.2003.00181.x

[mbo31174-bib-0052] Troccaz, M., Starkenmann, C., Niclass, Y., van de Waal, M., & Clark, A. J. (2004). 3‐Methyl‐3‐sulfanylhexan‐1‐ol as a major descriptor for the human axilla‐sweat odour profile. Chemistry & Biodiversity, 1, 1022–1035. 17191896 10.1002/cbdv.200490077

[mbo31174-bib-0053] van Herreweghen, F., Amberg, C., Marques, R., & Callewaert, C. (2020). Biological and chemical processes that lead to textile malodour development. Microorganisms, 8(11), 1709. 10.3390/microorganisms8111709 PMC7692034 33142874

[mbo31174-bib-0054] Villalaín, J., Mateo, C. R., Aranda, F. J., Shapiro, S., & Micol, V. (2001). Membranotropic effects of the antibacterial agent Triclosan. Archives of Biochemistry and Biophysics, 390, 128–136. 11368524 10.1006/abbi.2001.2356

[mbo31174-bib-0055] Zeng, X. N., Leyden, J. J., Lawley, H. J., Sawano, K., Nohara, I., & Preti, G. (1991). Analysis of characteristic odors from human male axillae. Journal of Chemical Ecology, 17, 1469–1492. 24257805 10.1007/BF00983777

